# The effect of JQ1 systemic administration on oxidative stress and apoptotic markers in renal ischemic reperfusion injury in a rat model

**DOI:** 10.25122/jml-2022-0287

**Published:** 2023-05

**Authors:** Saba Sahib Younis, Fadhaa Abdul Ameer Ghafil, Sahar Majeed, Najah Rayish Hadi

**Affiliations:** 1. Al-Sadr Medical City, Al-Najaf Health Directorate, Al Najaf Al-Ashraf, Iraq; 2. Department of Pharmacology and Therapeutics, Faculty of Medicine, University of Kufa, Najaf, Iraq

**Keywords:** JQ1, ischemia reperfusion injury, caspase-3, FOXO4, TNF-alpha, PI3K/AKT

## Abstract

This study aimed to investigate the effects of JQ1 in a renal ischemia-reperfusion (IR) rat model. Twenty-four adult male Wistar Albino rats were randomly divided into four equal groups. The sham group underwent laparotomy without ischemia-reperfusion induction. The control group experienced bilateral renal ischemia for 30 minutes, followed by a 2-hour reperfusion period. The vehicle group (IR group + DMSO) and JQ1 group (same as in control IR + 25 mg/kg JQ1). Kidney and blood samples were collected 2 hours after reperfusion. Blood samples were used to analyze serum creatinine and blood urea nitrogen levels. Renal tissue was assessed for TNF-alpha, caspase-3, FOXO4, PI3K/AKT signaling pathway, and histological analysis. The control group exhibited significantly higher serum creatinine, blood urea nitrogen, caspase-3, TNF-alpha, and FOXO4 levels in renal tissue compared to the sham group. Additionally, the PI3K/AKT signaling pathway was significantly decreased in the control group. Histopathological examination revealed severe kidney damage in the control group compared to the sham group. In rats treated with JQ1, serum creatinine, BUN, caspase-3, TNF-alpha, and FOXO4 levels in renal tissue significantly improved. The PI3K/AKT signaling pathway was substantially increased (p-value 0.01) compared to the Vehicle and Control groups. The tubular severity score was also significantly reduced in the JQ1-treated groups compared to the Control and Vehicle groups. In conclusion, JQ1 significantly ameliorated renal ischemia-reperfusion injury in rats by suppressing apoptosis and inflammatory pathways.

## INTRODUCTION

Acute kidney injury (AKI), also known as acute renal failure (ARF), refers to the sudden and often reversible loss of kidney function, as indicated by the glomerular filtration rate (GFR)1. Although blood concentrations of urea nitrogen (BUN) or creatinine may remain within normal ranges shortly after a renal injury, ischemia-reperfusion injury (IRI) is a two-step process that starts with reduced blood flow to the kidneys (RBF) and concludes with the restoration of blood flow. Clinical causes of IRI include infarction, sepsis, and organ transplantation [2]. The severity of IRI damage is determined by the tissue's metabolic needs, the mechanism and amount of reperfusion, as well as the length of the ischemic insult [3]. Renal ischemia-reperfusion injury activates multiple inflammatory pathways that contribute to further kidney damage. Tumor necrosis factor (TNF) plays a crucial role in systemic inflammation and is a key mediator in the acute phase response, acting as an intracellular signaling protein (cytokine). TNF is primarily produced by activated macrophages.

Reactive oxygen species are high-energy molecules that interact with an electron not attached to most outer orbitals. These free radicals damage cell membranes and trigger a signaling cascade inside cells. At some point during ischemia-reperfusion, inhibiting this process or blocking free radical formation may be a strategy for protecting tissues. FoxO4 (AFX) is a transcription factor belonging to the Forkhead (Fox). It was first discovered as a tumor suppressor but has since been connected to cell death in other metabolic and ischemia diseases, such as ischemic limbs, diabetes retinopathy, and diabetic nephropathy [4]. Apoptosis causes the activation of caspases, which controls all the morphological modifications that characterize this kind of cell death. Cysteine proteases, called caspases, exclusively consume aspartic acid residues as food. Caspases are created as inert dimers lacking protein-interaction domains [5]. Caspase-3, the main apoptosis effector, is upregulated in the early phases of IRI-induced AKI in renal tubular and microvascular endothelial cells [6]. Numerous cellular processes, including cell survival, reproduction, metabolism, neurology, motility, and cancer growth, are regulated by the PI3K/Akt signaling pathway. The lipid kinase family includes PI3K, which can phosphorylate the inositol ring 3'-OH group in the inositol phospholipids in plasma membranes. A well-studied family of signal transduction enzymes that control cellular activation, inflammation, and death includes PI3K and its downstream effector Akt [7]. JQ1 is one of the first and best-studied BRD4 inhibitors. In pancreatic, lung, prostate, breast, and acute myeloid leukemia models, JQ1 has been demonstrated to help reduce tissue damage [8]. Additionally, in a mouse model of myocardial infarction, JQ1 decreased cardiomegaly, pulmonary congestion, hypertrophy, and fibrosis. [9]. Furthermore, it has also been shown to improve kidney function, decrease tubular apoptosis in nephrotoxicity [10], enhance renal function, and decrease ER stress and apoptosis in a kidney IRI model [11].

## MATERIAL AND METHODS

The study was conducted at the Middle Euphrates Unit for Cancer Research and the Department of Pharmacology and Therapeutics at the Faculty of Medicine, University of Kufa. The Committee Center for Bioethics at the University of Kufa, represented within the Faculty of Medicine, approved the study (Pharmacology and Therapeutics Department), and all procedures were conducted following the Committee's recommendations.

### Study design

Adult male Wistar Albino rats weighing 250–350 g and 16–24 weeks old were kept in an animal house at the College of Science, University of Kufa. The temperature was kept at 24±2°C, humidity at 60-65%, and a 12-hour light/dark cycle for two weeks before the procedure. All rats underwent midline laparotomies to remove the left kidney under general anesthesia, using intraperitoneal injections of 100 mg/kg ketamine and 10 mg/kg xylazine [12]. Rats were randomly divided into four groups, with six rats in each group:

1. Sham group: Rats were subjected to the previously mentioned procedures without any systemic administration and induction of renal ischemia;
2. Control group: Rats in the control group had bilateral renal ischemia for 30 minutes, followed by two hours of reperfusion [13];
3. Vehicle group: Rats were intraperitoneally injected with DMSO about 30 minutes before renal ischemia, serving as a vehicle for JQ1, followed by 2 hours of reperfusion (Med Chem express/USA);
4. JQ1 pretreatment group: Rats received intraperitoneal JQ1 administration at a dose of 25 mg/kg for seven days before undergoing 30 minutes of renal ischemia and 2 hours of reperfusion [14].

### Experimental procedure

Rats were sedated using ketamine and xylazine hydrochloride, placed on their backs, and secured their limbs and tails with medical plaster. A midline laparotomy incision was made to expose the abdomen and right and left renal pedicles. To prevent dehydration, 1 cc of isotonic sodium chloride 0.9% solution warmed to 37°C was applied to the abdominal area. After two hours of reperfusion, the animals were euthanized by performing a cardiac puncture, and the left kidney was collected for laboratory testing and histological analysis [15]. It has been shown that kidney damage occurs when ischemia lasts longer than 20 minutes. Irreversible damage can result from more than 40 minutes of ischemia followed by reperfusion, while ischemia lasting between 20 and 40 minutes may be reversible [16].

### JQ1 preparation

The pure JQ1 powder from Solarbio Company was obtained and dissolved in a standard vehicle using a laboratory vibrator device. The vehicle solution was prepared by combining four solutions in different proportions (10% DMSO, 40% PEG300, 5% Tween 80, and 45% saline). The dosage of the drug used in the study was 25 mg/kg of rat weight, and it was administered via intraperitoneal injection [14].

### Tissue preparation for histopathology

The left kidney tissue sample was washed in xylene, embedded in a paraffin block, fixed in 10% formalin, and dehydrated in an alcohol series. Tissue slide sections were cut horizontally to a thickness of approximately 5 micrometers and stained with Hematoxylin and Eosin dye before being sent to a histopathologist for histological evaluation. After examining the slides at a magnification of X40, the degree of renal damage was assessed [17]. The histological alterations score was calculated as a percentage of renal tubular damage as follows:

- Score 0: Represents normal tissue;
- Score 1: Represents < 25% of damaged tubules;
- Score 2: Represents 25% - 50% of damaged tubules;
- Score 3: Represents 50% - 75% of damaged tubules;
- Score 4: Represents > 75% of damaged tubules [18].

### Serum assays

Approximately 3.5-5ml of blood was collected from the heart of each rat at the end of the surgical procedure and placed in a gel tube without anticoagulant. The gel tubes were labeled, placed in a rack, and centrifuged at 3000 rpm for 15 minutes. The resulting serum was used to determine the levels of urea and creatinine.

### Tissue preparation for TNFα, FOXO4, PI3K/AKT, CASPASE-3 measurement using ELISA

At the end of the surgical procedure, the left kidney was removed and rinsed with ice-cold normal saline to remove any blood clots and then dissected into two parts. One section was taken, and used a high-intensity ultrasonic liquid processor to homogenize in 1:10 (w/v) phosphate-buffered saline containing 1% Triton X-100 and a protease inhibitor cocktail [12]. At 4°C, the homogenate was centrifuged for 20 minutes at 14000 rpm [19]. The supernatant was collected for ELISA method detection of TNF-alpha FOXO4, Caspases 3, pi3k/AKT.

### Measurement of study parameters

The following steps were taken to measure the inflammatory (TNF-α), apoptotic (caspase-3), oxidative stress (FOXO4), and PI3K/AKT signaling pathway markers using the ELISA method. All reagents, standard solutions, and samples were prepared as instructed and brought to room temperature before use. The assay was performed at room temperature. The number of strips required for the assay was determined, and the strips were inserted into the frames for use. Unused strips were stored at 2-8°C. Next, 50 μl of the standard was added to the standard well. Antibody was not added to the standard well because the standard solution already contained a biotinylated antibody. For the sample wells, 40μl of the sample was added, followed by 10μl of anti-TNFα, anti-caspase3, anti-FOXO4, and anti-pi3k/AKT antibodies. Then, 50μl of streptavidin-HRP was added to both the sample and standard wells (but not the blank control well), and the plate was mixed well and covered with a sealer. It was then incubated for 60 minutes at 37°C. After removing the sealer, the plate was washed 5 times with wash buffer. The wells were soaked with at least 0.35 ml wash buffer for 30 seconds to 1 minute for each wash. For automated washing, all wells were aspirated and washed 5 times with wash buffer, overfilling wells with wash buffer. The plate was then blotted onto paper towels or other absorbent material. To each well, 50μl of substrate solution A was added, followed by 50μl of substrate solution B. The plate was then incubated, covered with a new sealer, for 10 minutes at 37°C in the dark. Finally, 50μl of Stop Solution was added to each well, and the blue color changed into yellow immediately. The optical density (OD value) of each well was determined using a microplate reader set to 450 nm within 10 minutes after adding the stop solution. The unit of tissue TNFα and other ELISA results is ng/ml according to the manufacturer's instructions.

### Statistical analysis

Statistical analysis was performed using Graph Pad Prism version 9. The data was presented s the mean ± standard error of the mean, and for multiple comparisons, ANOVA and the LSD posthoc test were used. The histopathological data were analyzed using Mann Whitney U and Kruskal-Wallis test. A p-value of less than 0.05 was considered statistically significant for each test.

## RESULTS

### JQ1 ameliorate renal function

**Figure 1.**
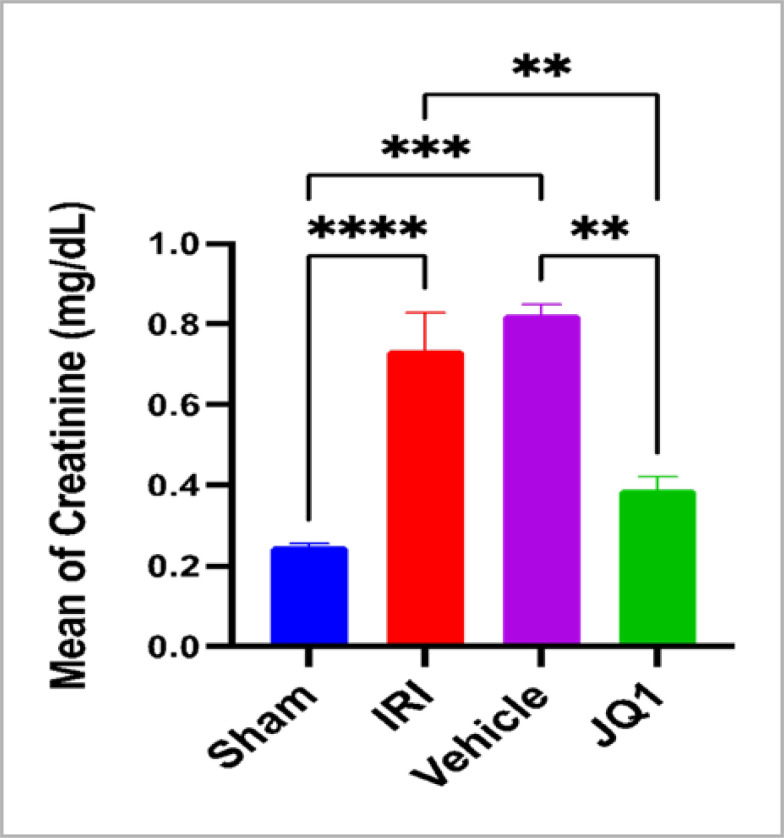
Mean serum creatinine levels (mg/dl) SEM across groups (n=6 rats per group). **Significant difference JQ1 vs. vehicle, JQ1vs IRI, p-value<0.01. ***Significant difference vehicle vs. sham, p-value<0.001. ****Very high significant difference sham vs. IRI, p-value<0.0001

**Figure 2.**
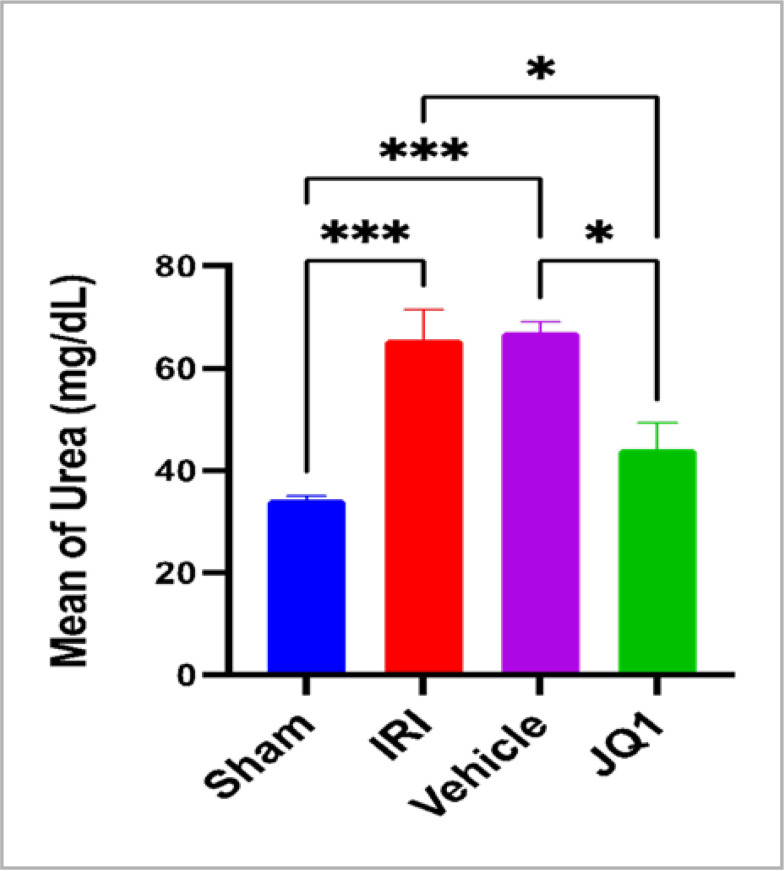
Mean serum level of urea (mg/dl) ± SEM across groups (n=6 rats per group). *Significant difference JQ1 vs. vehicle, JQ1 vs. IRI, p-value<0.01. ***High significant difference sham vs. IRI, sham vs. vehicle, p-value<0.001

Blood urea and creatinine levels significantly increased in the control and vehicle groups compared to the sham group. However, JQ1 pretreatment resulted in significantly lower levels of both kidney function indicators when compared to the control and vehicle groups, as shown in Figures 1 and 2.

### JQ1 decreased inflammatory marker TNF-alpha in renal tissue

**Figure 3.**
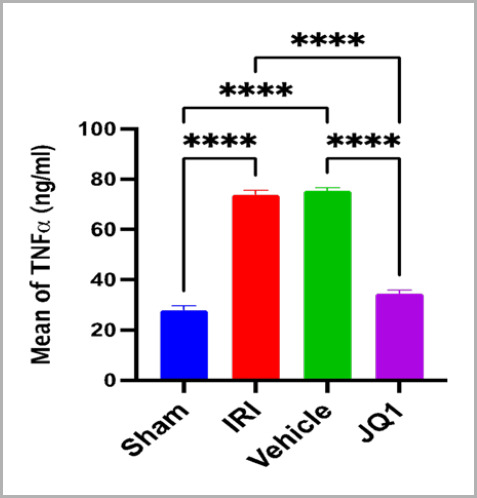
Mean renal tissue of TNFα (ng/ml) across groups (n=6 rats per group). ****Very high significant difference sham vs. IRI, sham vs. vehicle, JQ1 vs. IRI, JQ1 vs. vehicle, p-value<0.0001

Renal tissue levels of TNF-alpha were significantly higher in the control and vehicle groups than in the sham group. Compared to the control and vehicle groups, the 25mg/kg JQ1 pretreatment group had a significant reduction in the protein expression of the inflammatory mediator TNF-alpha (Figure 3).

### JQ1 reduced oxidative stress (FOXO4) in renal tissues

**Figure 4.**
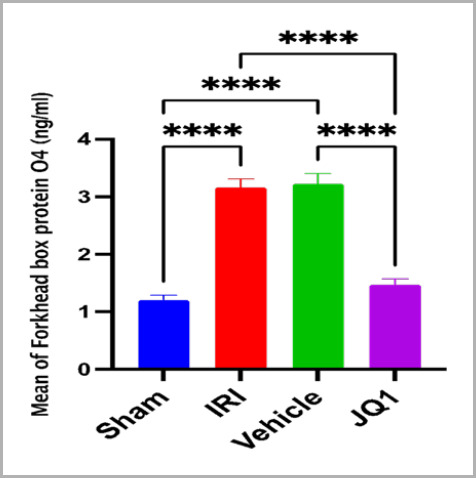
Mean FOXO4 concentration (ng/ml) across groups (n=6 rats per group). ****Mean highly significant difference between sham vs. IRI, sham vs. vehicle, JQ1 vs. IRI, JQ1 vs. vehicle, p-value<0.0001

Compared to the sham group, rats in the control and vehicle groups showed a significantly higher level of FOXO4 in their renal tissues. When compared to the control and vehicle groups, JQ1 pretreatment significantly lowered the renal tissue level of FOXO4 (Figure 4).

### JQ1 attenuated apoptotic marker caspase-3 in renal tissue

**Figure 5.**
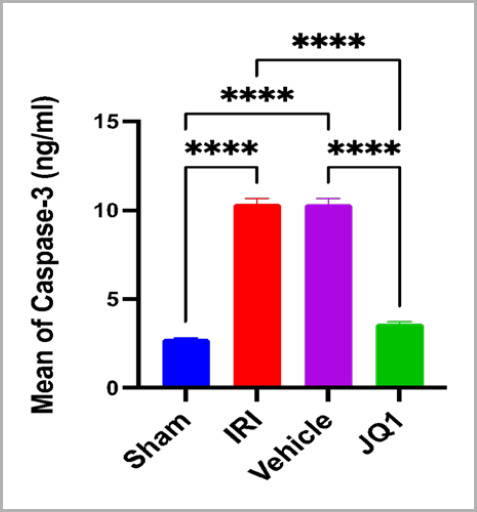
Mean caspases-3 (ng/ml) across groups (n=6 rats per group). ****Very high significant difference sham vs. IRI, sham vs. vehicle, JQ1 vs. IRI, JQ1 vs. vehicle, p-value<0.0001

Compared to the sham group, rats in the control and vehicle groups had significantly higher levels of caspase-3 in their renal tissue. However, the JQ1 pretreatment group had significantly lower levels of caspase-3 in their renal tissue compared to the control and vehicle groups (Figure 5).

### JQ1 upregulated the PI3K/AKT expression

**Figure 6.**
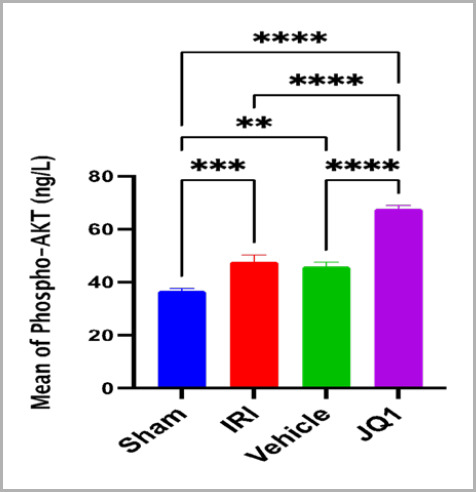
Mean P-AKT concentration (ng/ml) across groups (n=6 rats per group). **Mean significant difference sham vs. vehicle, p-value<0.01. ***Mean high significant difference sham vs. IRI, p-value<0.001

**Figure 7.**
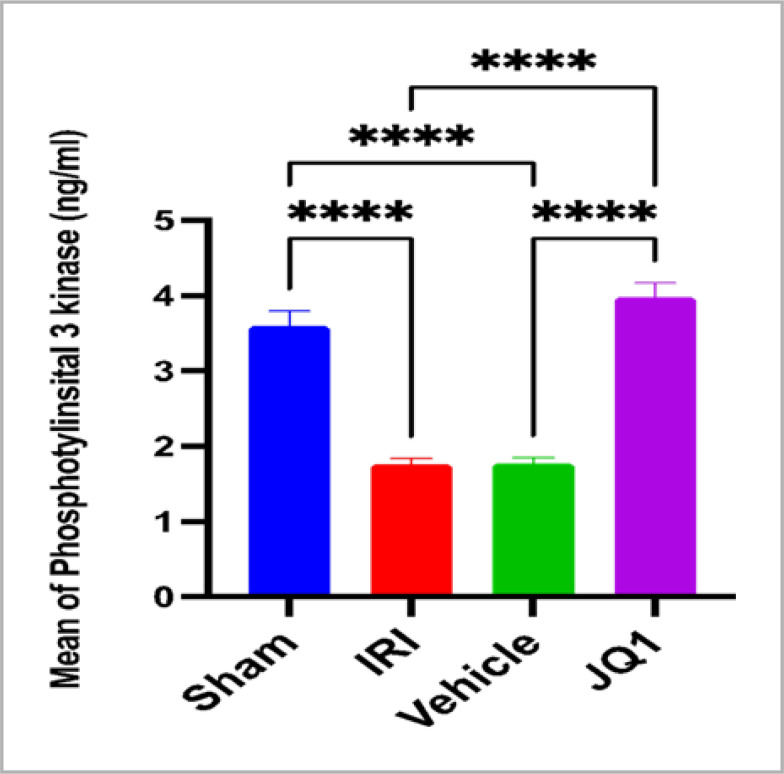
Mean PI3K levels (ng/ml) across groups at the end of the experiment (n=6 rats per group). ****Mean very high significant difference sham vs. IRI, sham vs. vehicle, JQ1vs IRI, JQ1vs vehicle, p-value<0.0001

The results revealed that compared to the control and vehicle groups, the levels of PI3K/AKT protein expression were significantly higher in the sham group. In contrast, JQ1 treatment significantly increased the expression of these proteins, as demonstrated in Figures 6 and 7.

### JQ1 minimized kidney injury

**Figure 8.**
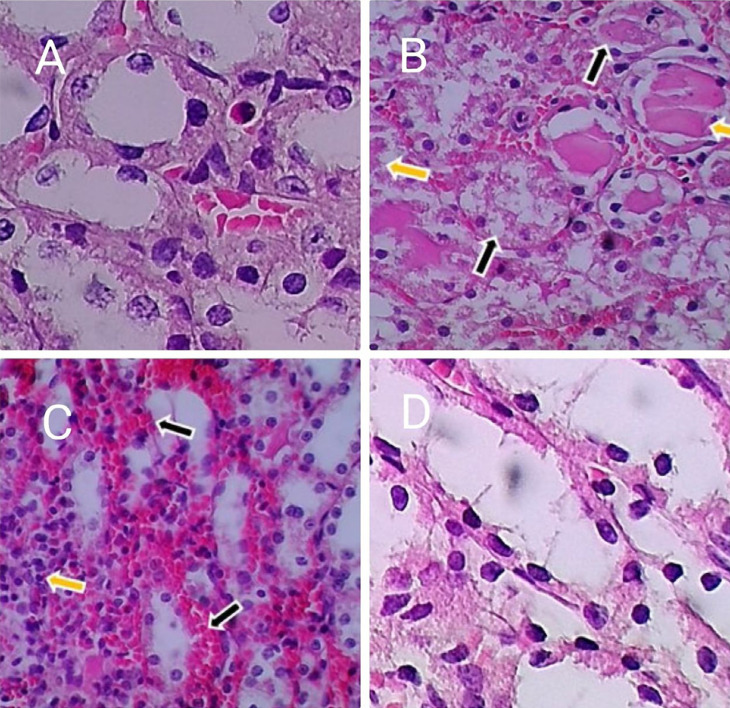
Renal segment from histopathological analysis. A: renal segment photomicrograph for the sham group, kidney of the rat with normal histology of mean intensity score of tubular damage in the sham group in the renal tubules H&E, 400x; B: photomicrograph shows the control group's renal portion: kidney of the rat with score 4 ischemic reperfusion damage of renal tubules. Increased cytoplasmic eosinophilia, cellular swell, loss of the brush border, and presence of eosinophilic casts, hemorrhage (black arrows) surrounded renal tubules and glomerulus, and occupied spaces of the necrotic renal tubule, H&E, 400X; C: renal segment photomicrograph for the vehicle group, kidney of the rat with score 4 ischemic reperfusion damage of renal tubules. Diffuse cellular swelling, increased cytoplasmic eosinophilia, loss of brush border, and hemorrhage/ necrosis of epithelial cells (black arrows) of renal tubules of cortex area, where the inflammatory exudate (yellow arrows) was observed in the lumen of renal tubules under necrosis, H&E 400X; D: renal segment photomicrograph for the JQ1 treatment group, kidney of the rat with score 1 ischemic reperfusion damage affect 25% of renal tubules cellular swelling, and increase cytoplasmic eosinophilia. H&E 400x.

The kidney in the sham group had modest tubular damage, according to histopathological analysis. In comparison to the control group, more damaged tubules and enlarged cells were seen in the control and vehicle groups. Compared to the control and vehicle groups, the JQ1 pretreatment group exhibited minimal histological alteration (Figure 8 A-D).

## DISCUSSION

The primary cause of acute kidney injury (AKI) is renal ischemia-reperfusion injury. In vivo, I/R-induced expression of the apoptotic and ERS proteins was reduced by BRD4 suppression, while FOXO4 was inhibited from producing ROS. This shows that BRD4 may be a therapeutic target and that JQ1 may be a potential drug for renal ischemia injury.

### Effect of JQ1 on urea and creatinine

The experimental results demonstrate that pretreatment with the selective BRD4 inhibitor JQ1 significantly reduces the levels of creatinine and urea in comparison to the levels in the control and vehicle groups after ischemia induction. This finding suggests that JQ1 has a protective effect on renal function indices (urea and creatinine) following renal ischemia-reperfusion injury (IRI) induction in a rat model. This result is consistent with previous studies, which have demonstrated that JQ1 treatment improves renal function, as indicated by urinary albumin to creatinine ratio and serum creatinine levels, and improves glomerular structural damage (such as extraciliary growth and fibrinoid necrosis) [20]. In a different study, JQ1 reversed renal illnesses (hyaline cast formation and renal tubule lysis) and improved renal function (measured by blood creatinine and BUN levels) in a model of acute kidney injury induced by the nephrotoxic drug cisplatin [20].

### Effect of JQ1 on inflammatory mediator TNF-alpha

This experimental study demonstrated that pretreatment with the BRD4 selective inhibitor JQ1 prior to the induction of ischemia might dramatically reduce the level of inflammatory cytokines (TNF) compared to those in the control and vehicle groups. This suggests that JQ1 has an anti-inflammatory impact on renal tissues that have experienced ischemia and reperfusion. This finding is consistent with previous research [21] that established that BRD4 inhibitors have anti-inflammatory activities in various preclinical tests. It suggests that there may be another mechanism underlying the anti-inflammatory effects of BRD4 inhibitors in renal damage. JQ1 treatment has also been shown to reduce renal inflammation and inhibit the NF-κB pathway in the UUO model by inhibiting the phosphorylation and acetylation of NF-κB, resulting in a decrease in the number of CD68+ cells (macrophages) present in the tubule interstitial [20].

### Effect of JQ1 on oxidative stress marker FOXO4

This experimental study demonstrated that JQ1 pretreatment prior to ischemia induction significantly reduced the levels of the oxidative stress marker FOXO4 in renal ischemic tissues compared to the levels in the vehicle and control groups. This result is consistent with previous research [22] that showed JQ1 decreased the levels of FoxO4 protein, its transcriptional activity, and the formation of ROS and H2O2, which was reported to lessen the oxidative stress induced by I/R damage.

### Effect of JQ1 on apoptotic marker caspase 3

The study showed a marked reduction in renal caspase-3 levels in the JQ1 treatment group compared to the control group. This finding suggests that JQ1 may reduce apoptosis in renal tissue following IRI. This result is consistent with a previous study [14], which found that IRI increased the transcription of Bax and caspase-3 while decreasing the expression of Bcl-2. Additionally, they found that pretreatment with JQ1 significantly reduced the expression of Bax and caspase-3 and increased the expression of Bcl-2 compared to the I/R group. However, their results were obtained through Western blot and immunohistochemical analysis.

### Effect of JQ1 on PI3K/AKT activation

This study found that JQ1 treatment significantly increased the renal PI3K/AKT level compared to the control group. These findings suggest that JQ1 activates the PI3K/AKT survival pathway to provide nephroprotection against renal IRI. This supports the findings of a previous study [14], which showed that JQ1 pretreatment increased and I/R inactivated the PI3K/AKT signaling pathway. Activation of the Akt signaling pathway has been shown to facilitate the renoprotective actions of extracellular hepatic cell growth factor (HGF) on the kidney during a simulation of ischemia-reperfusion damage [23]. Furthermore, some researchers suggest that Akt has a small-molecule regulation binding affinity that is near the active region of the kinase, making it an attractive candidate for structure-based drug development [24].

### Effect on JQ1 on renal parenchyma

This investigation revealed that pretreatment with the selective Brd4 inhibitor JQ1 prior to the onset of ischemia could considerably lessen the severity of kidney injury compared to the severity of renal injury in the control and vehicle groups. In this pretreatment group, the mean of the proportional damage indicated mild to severe renal injury. Renal tissues were normal in the Sham group. However, kidneys in the I/R group exhibited acute tubular damage in the proximal tubules, including tubular dilatation and loss of the brush border. JQ1 protected the tubular epithelium from swelling and loss of the brush border [11].

## CONCLUSION

JQ1 efftively reduced renal ischemia reperfusion damage in rats via apoptosis and inflammatory pathway suppression.
